# An Undergraduate Student‐Led Neuroscience Outreach Program Shows Promise in Shifting Teen Attitudes About Drugs

**DOI:** 10.1111/mbe.12261

**Published:** 2020-10-04

**Authors:** Nina T. Lichtenberg, Andrew B. Thompson, Martin Y. Iguchi, Christopher J. Evans, Rafael Romero‐Calderón

**Affiliations:** ^1^ Department of Psychology UCLA USA; ^2^ Drug Policy Research Center RAND Corporation USA; ^3^ Brain Research Institute UCLA, Gonda (Goldschmied) Neuroscience and Genetics Research Center USA; ^4^ Hatos Center for Neuropharmacology Semel Institute for Neuroscience and Human Behavior, UCLA USA; ^5^ Interdepartmental Program for Neuroscience UCLA, Gonda (Goldschmied) Neuroscience and Genetics Research Center USA

## Abstract

Drug Outreach, Promoting Awareness (DOPA) is an undergraduate outreach program for local high school students designed to convey the neurobiological basis, risks, and addictive potential of commonly abused drugs. Here we describe DOPA and evaluate the program, including its impact on high school student attitudes about drug harm risk and addiction. Undergraduate neuroscience students versed in the neurobiology, physiology, and policy of drugs are trained in active learning methods, enabling them to create engaging and interactive classroom‐based educational materials. Survey results showed that participation in DOPA increased high school student perceptions of the addictive potential and harm risk of drugs, which studies have shown to be inversely correlated with drug‐taking. High school students also responded positively to the interactive nature of the program. These findings demonstrate how extensively trained undergraduates who are close peers to high school students can effectively lead science outreach initiatives and shift adolescent attitudes about drugs.

## Abstract 2

**Lay abstract:** Here we describe a novel high school drug outreach program led by undergraduate neuroscience students. We investigated how the program impacted high school student attitudes about the harm risks and addictive potential of commonly used drugs of abuse. The program was valued highly by the undergraduates, and surveys showed increased awareness of the health risks of several drugs among high school students. The findings demonstrate how using an interactive, peer‐led approach to drug outreach can positively impact adolescents.

The importance of neuroscience outreach has been widely acknowledged by federal funding agencies (D. P. Friedman, 2008; Leshner, 2007) and professional societies, such as the Society for Neuroscience (Cameron & McNerney, 2006; McNerney, Chang, & Spitzer, 2009). Broadly speaking, community outreach engages the lay public in scientific discovery and dialogue, and benefits scientists by enhancing their communication skills (Varner, 2014). Recognizing the value of outreach, many higher education institutions (ourselves included) have developed undergraduate student‐led neuroscience outreach programs (Butcher, Do, Wensler, Shah, & Thorne, 2010; Deal, Erickson, Bilsky, Hillman, & Burman, 2014; Edlow, Hamilton, & Hamilton, 2007; Gittis, 2010; Romero‐Calderón et al., 2012; Stevens, 2011; Vollbrecht, Frenette, & Gall, 2019; Yawson et al., 2016). Collectively, these programs are centered on educating K‐12 students in broad neuroscience themes, such as neuroanatomy, sensory perception, cognition, mental health, and drugs.

Interestingly, apart from a few initiatives (Epstein, Noel, Finnegan, & Watkins, 2016; Hamrick, Harter, Fox, Dhir, & Carrier, 2019; Surratt & Desselle, 2004; Yim, Esperanza, & Puder, 2018), science outreach efforts rarely focus on drugs. Such outreach programs are valuable because they provide adolescents with science‐based information about drugs, while also engaging them in real‐world issues at the interface of science and society. Indeed, drug misuse is a major ongoing public health crisis in the United States. In 2018, there were over 67,000 drug overdose deaths–including those involving illicit drugs and synthetic opioids (Ahmad, Rossen, Spencer, Warner, & Sutton, 2018; Hedegaard, Miniño, & Warner, 2020). Furthermore, evidence suggests that most adults with a substance use disorder initiate drug use in adolescence: the median age for initiation is 16 years, with 50% of cases beginning between ages 15 and 18 (Jordan & Andersen, 2017), highlighting the importance of interventions during adolescence.

Capitalizing on our experience running effective K‐12 neuroscience outreach courses (Romero‐Calderón et al., 2012; Saravanapandian et al., 2019), we created Drug Outreach, Promoting Awareness (DOPA). Founded in 2013 by the University of California, Los Angeles (UCLA) Brain Research Institute, in collaboration with the UCLA Undergraduate Interdepartmental Program for Neuroscience and the UCLA Hatos Center for Neuropharmacology, DOPA provides scientifically accurate information about the health risks and public policy behind drugs. Studies suggest that school‐based drug prevention programs that are targeted, evidence‐based, interactive, and peer‐led are effective at reducing drug misuse (MacArthur, Harrison, Caldwell, Hickman, & Campbell, 2016; Robertson, David, & Rao, 2003; Tobler et al., 2000). Notably, active learning strategies have been adopted by several empirically proven mainstream youth drug prevention programs, including Communities that Care (Hawkins et al., 2009), Project Towards No Drug Abuse (Sussman, Dent, & Stacy, 2002), and other community‐based models (Moore, Karpinski, & Tsien, 2018; Robertson et al., 2003).

DOPA employs an interactive, hands‐on educational approach to convey that: (1) Illicit drugs have unreliable and sometimes toxic constituents (Solimini et al., 2017); (2) Drugs can have major physiological and psychological effects both acutely and during abstinence (National Institute on Drug Abuse, 2020); (3) Polydrug use can have unanticipated consequences (Coffin et al., 2003; Kandel, Hu, Griesler, & Wall, 2017); and (4) Addiction is comorbid with other mental health disorders, such as depression and anxiety (Conway, Swendsen, Husky, He, & Merikangas, 2016; Santucci, 2012). Importantly, we promote an honest, candid approach to drug awareness that allows high school students to discover and evaluate the risks for themselves.

Surveys administered to participating high school students before and after classroom visits showed that our novel program increased perceptions of the harm risk and addictive potential of most drugs in the curriculum, particularly for those who were unfamiliar with certain drugs.

## METHODS

### The UCLA DOPA Program Undergraduate Course Curriculum

**Fig 1:**
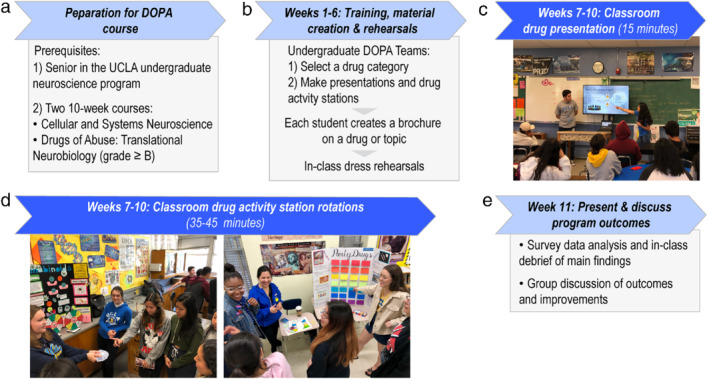
Timeline depicting the format of the University of California, Los Angeles (UCLA) Drug Outreach, Promoting Awareness (DOPA) outreach course. (a) Requirements for enrollment and participation in the DOPA course, which include two prerequisite courses titled M101A Neuroscience: From Molecules to Mind—Cellular and Systems Neuroscience (5 units) and C177 Drugs of Abuse: Translational Neurobiology (4 units). (b) Structure and primary workshop activities during the first 6‐week module of the 10‐week course. (c) Classroom visits during the second 4‐week module of the course. Example of a 15‐min presentation by two undergraduates explaining the chemical contents of nicotine vaporizers. (d) Left: Undergraduate students use a hands‐on activity with playing cards and spin wheels to demonstrate the unknown health consequences of using cannabinoids and spice/ K2. Right: Undergraduate students use a board game and Q&A cards to illustrate that it is often impossible to know the identity of chemical compounds found in most party drugs (e.g., ecstasy or MDMA). (e) The graduate student teaching assistant analyzes the current year's survey data and presents main findings to the undergraduate class. Undergraduate students then discuss the results with course instructors and reflect on the course and their experience.

To facilitate peer‐led instruction, our course (Supporting File 1) trains senior UCLA undergraduates majoring in neuroscience in giving accurate, interactive presentations and activity stations to high school students (grades 9–12; ∼14‐ to 18‐year‐olds). The course (formally named Drug Abuse and Society: Conveying Concepts to High School Students) can enroll up to 12 undergraduates and meets once a week for a three‐hour block. It is instructed by two faculty (providing drug pharmacology and pedagogical expertise) and a graduate student teaching assistant. Undergraduates are expected to utilize the knowledge gained from two prerequisite courses to develop educational materials for high school students (Figure 1a). Broadly, the 10‐week DOPA course is divided into two modules: a 6‐week component to generate the content of their presentations, activities, and brochures (Figure 1b), and a 4‐week component focused solely on school visits (Figure 1c and d). A debrief is held during finals week to review survey results (Figure 1e). Weekly class meetings are designed as workshops in which undergraduate students and instructors freely discuss concepts, practice active learning methods, review youth drug trends, brainstorm hands‐on activities, clarify misconceptions, and exchange ideas about drugs. We focus on drugs that high school students are most commonly exposed to (Miech et al., 2019) and aggregate them into the following categories: (1) Cannabinoids (THC, CBD and synthetic cannabinoids); (2) Legal recreational drugs (alcohol and nicotine products); (3) Prescription drugs (opioids and stimulants such as Adderall®); and (4) Party and/ or date‐rape drugs (substances such as ecstasy; psychedelics; ketamine; and Gamma‐hydroxybuterate).

Teams of 2–3 undergraduate students (DOPA Teams) create a 15‐min oral presentation to introduce the program along with their drug category and a hands‐on activity to teach about the effects and/ or risks of specific drugs. During the first module (Figure 1b), undergraduate students are coached in oral communication skills including presentation design, voice projection, audience engagement, and conveying facts accurately. Before classroom visits, each team performs two rehearsals of their presentation and activity in front of classmates and course instructors. These practice runs allow us to check for factual accuracy and presentation clarity as well as to offer improvements in lecturing style.

Lastly, each undergraduate student is mentored in effective science writing by designing an informational brochure on one drug or topic within their category to distribute in classrooms (Supporting File 2). Overall, this group learning encourages students to think creatively and to work cooperatively to design engaging lessons. We openly provide all DOPA educational materials online (http://www.bri.ucla.edu/outreach/drug‐abuse‐and‐society).

### The UCLA DOPA Program High School Classroom Visits

During the second course module (Figure 1c and d), DOPA visits 5–8 classrooms in 3–4 high schools in the greater Los Angeles area, including private, public, and charter schools within the public Los Angeles Unified School District (LAUSD) (Supporting File 3). Participating high schools are selected by proximity to campus and expressed interest in drug education from an updated list of schools that have regularly participated in UCLA outreach activities over the past decade (Saravanapandian et al., 2019).

Each visit lasts about 50 min and requires the participation of all enrolled undergraduate students. The session starts with a 15‐min presentation by one undergraduate team introducing the program, how drugs target neurotransmission and a preselected drug category. During the remaining 35–45 min, high school students rotate through four drug activity stations, one for each drug category. The presenters provide an activity that illustrates some aspect of the drugs covered in the presentation while the other 2–3 groups run activity stations focused on the remaining three drug categories. When time and personnel permit, we include a brain anatomy station to teach about areas of the brain affected by drugs and addiction.

Open discussion and questions by high school students and teachers are highly encouraged and undergraduate students are coached during rehearsals to approach discussion points in an honest, professional manner. Importantly, at the activity stations, high school students and teachers can interact one‐on‐one with undergraduates and ask them additional questions about drugs, the brain, and research or college life.

### Survey Evaluations

To evaluate the program's immediate impacts, we administered online pre‐ and postvisit surveys (Supporting Files 4 and 5) to participating high school students (previsit *n* = 428; postvisit *n* = 234) via Google Forms (http://forms.google.com). Participation was strictly voluntary and anonymous. Previsit surveys were administered to obtain high school students' baseline perceptions of drugs. Postvisit surveys were administered 1 to 2 weeks after each visit so that we could appropriately measure consolidated attitudes or perceptions about the material (Soderstrom & Bjork, 2015). Due to the difficulty of administering surveys in a busy classroom setting, the exact timing of each survey was determined by the host teacher. We matched pre‐ and postvisit responses by asking participants to tell us their gender, the first three letters of their mother's maiden name, their favorite color and their favorite number.

Surveys included the following questions for each of nine drugs: (1) Whether they had heard of the drug; (2) How common the drug was amongst their peers; (3) How harmful they thought the drug was; and (4) How addictive they thought the drug was. Question 1 was a “yes” or “no” while questions 2–4 used a 5‐point Likert‐type scale (1 = *low*, 5 = *high*) (Birkett, 1986). We also asked high school students several open‐ended questions, which included: (1) If they have any questions or drugs they would like to learn about; (2) What they would do if someone important to them asked about a treatment for an addiction; and (3) Their interest level in the DOPA visit. For the postvisit survey, participants were asked; how valuable the information was for each drug, how valuable the DOPA program was overall, and what they found most interesting.

### Statistical Analyses

Approximately 73.5% of individual postvisit responses were matched to previsit responses (*n* = 540 total participants, *n* = 428 total previsit responses, *n* = 234 total postvisit responses, *n* = 172 matched responses). Given the ordinal nature and non‐normality of the data, nonparametric Friedman tests were used to determine differences between pre‐ and postvisit ratings (M. Friedman, 1937). Post‐hoc analyses were performed with Wilcoxon paired *t*‐tests to compare matched ratings within each group of interest (Meek, Ozgur, & Dunning, 2007). Mann–Whitney unpaired *t*‐tests were used when comparing changes in individual drug perceptions between sexes. Significance was assessed at the *p* < .05 level. Effect sizes were calculated with the formula *r* = Z/√ *N* and interpreted according to Cohen's Pearson *r* benchmarks (Cohen, 1988; Fritz, Morris, & Richler, 2012). Prism version 8.0 (GraphPad, San Diego, CA) and SPSS version 23 (SPSS, Chicago, IL) were used for data analysis.

### Ethics Statement

The UCLA Institutional Review Board (IRB) determined that the evaluation of our educational outreach activities did not meet the definition of human subjects research as defined by federal regulation for human subject protections [45 CFR 46.102(d)]. No personal identifying information was gathered and high school students (or a parent/ legal guardian), as well as teachers signed a waiver of liability and media release consent form.

## RESULTS

Over 3 years (2016–2018), 24 UCLA neuroscience undergraduate students completed the DOPA course and visited 18 classrooms in six Los Angeles area schools, instructing over 540 high school students in total.

### Survey Results: Perceptions of Harm Risk and Addictive Potential of Drugs

**Fig 2:**
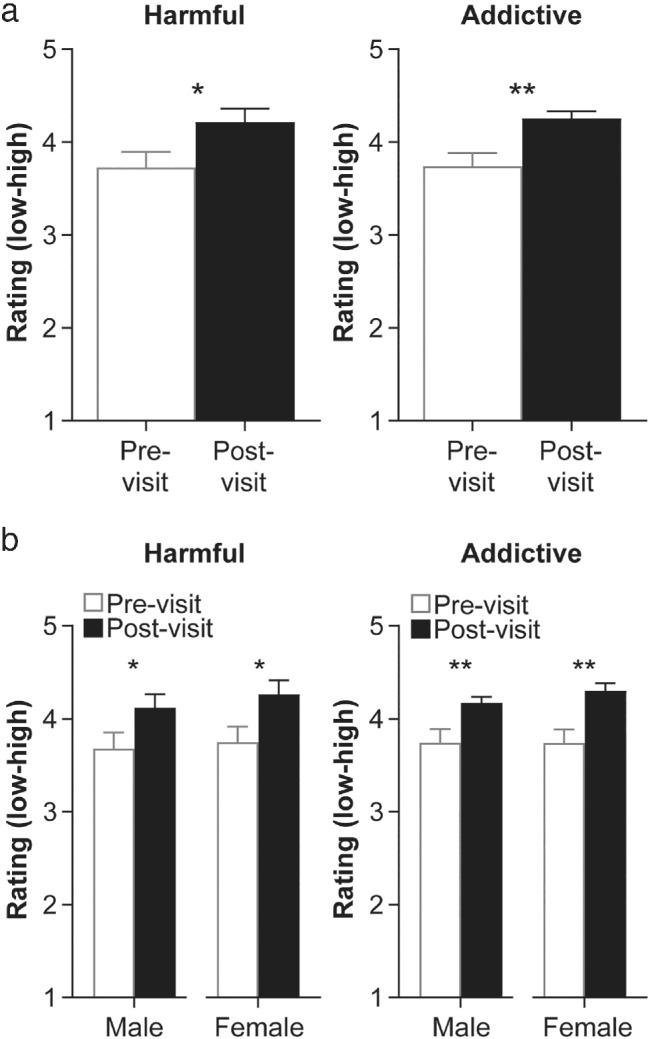
Changes in mean ratings of harm and addictive potential of drugs of abuse overall and across genders. (a) Average (±SEM) of matched pre‐ and postvisit ratings (1 = *low*, 5 = *high*) of harm risk (left) and addictive potential (right) of all nine drugs combined (*n* = 172). (b) Average (±SEM) pre‐ and postvisit ratings of harm risk (left) and addictive potential (right) of all drugs combined for male and female high school students (*n* = 60 male, *n* = 112 female). Nonparametric Friedman tests conducted; *ns p* > .05; *p < .05, **p < .01, ***p < .001 indicate levels of significance.

Based on past research showing an inverse correlation of drug‐taking with perceived risk (Szalay, Inn, Strohl, & Wilson, 1993), we conducted an initial assessment of perceptions of harm risk and addiction as preliminary markers of program impact. Combining survey data from all 3 years (2016–2018) revealed that the DOPA program significantly and consistently changed high school students' perceptions of the risks of harm and addictive potential of drugs overall. When comparing matched pre‐ and postvisit ratings for all nine drugs collectively, a Friedman test revealed a significant increase in both harmfulness, *χ* ^2^(1) = 5.44; *p* = .020, and addictiveness, *χ* ^2^(1) = 9.00; *p* = .003, ratings (Figure 2a). Given the overall difference in ratings, we then compared changes in pre‐ and postvisit ratings for each drug individually. We found that the DOPA program impacted high school students' perceptions of both harmfulness and addictiveness for most drugs included in the program. Wilcoxon paired *t*‐tests revealed that harmfulness ratings significantly increased for all drugs except for alcohol, cigarettes, and marijuana, although the effect sizes for some drugs (i.e., ecstasy, mushrooms, and opioids) were small (Table 1). Similarly, addictiveness ratings significantly increased after DOPA for all drugs, except for cigarettes (Table 2). However, the effect sizes of addictiveness rating comparisons for alcohol, marijuana, and a few other drugs were also small, possibly reflecting our inability to shift attitudes about these more common drugs.

**Table 1:** Average Harmfulness Ratings for Nine Individual Drugs

	Mean rating (*SD*)				
Drug	Previsit	Postvisit	*p*	Effect size^a^	Unfamiliar (%)
Alcohol	4.08 (0.94)	4.07 (0.94)	.987	0.00	0 (0%)
Cigarettes	4.54 (0.79)	4.58 (0.75)	.596	0.03	0 (0%)
Marijuana	3.19 (1.24)	3.23 (1.18)	0.934	0.00	2 (1.2%)
Ecstasy	4.26 (1.02)	4.61 (0.66)	<.001	0.23	17 (9.9%)
Mushrooms	3.97 (1.08)	4.36 (0.89)	<.001	0.26	32 (18.6%)
Opioids	3.54 (1.21)	4.16 (0.96)	<.001	0.28	36 (20.9%)
Stimulants	3.11 (1.24)	3.96 (0.93)	<.001	0.37	44 (25.6%)
Spice	3.48 (1.23)	4.56 (0.69)	<.001	0.47	85 (49.4%)
GHB	3.35 (1.35)	4.40 (0.82)	<.001	0.42	96 (87.3%)

*Note*. *n* = 172 for all drugs except GHB (*n* = 110).

a Effect size *r*.

**Table 2:** Average Addictiveness Ratings for Nine Individual Drugs

	Mean rating (*SD*)				
Drug	Previsit	Postvisit	*p*	Effect size^a^	Unfamiliar (%)
Alcohol	4.01 (1.06)	4.27 (0.86)	<.001	0.20	0 (0%)
Cigarettes	4.56 (0.78)	4.66 (0.67)	.162	0.08	0 (0%)
Marijuana	3.60 (1.16)	3.83 (1.10)	.042	0.11	2 (1.2%)
Ecstasy	4.09 (1.10)	4.41 (0.86)	<.01	0.20	17 (9.9%)
Mushrooms	3.74 (1.10)	4.22 (0.90)	<.001	0.27	32 (18.6%)
Opioids	3.74 (1.28)	4.38 (0.89)	<.001	0.32	36 (20.9%)
Stimulants	3.22 (1.24)	4.09 (0.97)	<.001	0.38	44 (25.6%)
Spice	3.39 (1.23)	4.28 (0.91)	<.001	0.39	85 (49.4%)
GHB	3.31 (1.28)	4.15 (0.96)	<.001	0.36	96 (87.3%)

*Note*. *n* = 172 for all drugs except GHB (*n* = 110).

a Effect size *r*.

Despite a balanced high school student sex distribution at all the schools we visited (average total school enrollment was 49.6% and 50.4% for males and females respectively) and a representative distribution in our classrooms, our survey data suggested a strong sampling bias. Indeed, we noticed a marked asymmetrical sex ratio of survey participants (males: 35%; females: 65%), not unlike what has been previously measured (Porter & Whitcomb, 2005; Sax, Gilmartin, Lee, & Hagedorn, 2008), so we checked to see if our results were sex biased. After segregating the data by sex, separate Friedman tests confirmed that the program significantly increased harmfulness and addictiveness ratings of drugs overall by comparable magnitudes for both male (harmfulness: *χ* ^2^(1) = 5.44, *p* = .020; addictiveness: *χ* ^2^(1) = 8.00, *p* = .005), and female (harmfulness: *χ* ^2^(1) = 5.44, *p* = .020; addictiveness: *χ* ^2^(1) = 9.00, *p* = .003), high school students (Figure 2b), indicating that we could pool data across sexes. It should be noted however, that males rated alcohol as significantly less addictive than females before the visit (Mann–Whitney unpaired *t*‐test, mean rating ± *SD*; males: 3.75 ± 1.19; females: 4.14 ± 0.97, *p* = .037), but showed no statistical differences postvisit (addictiveness mean rating ± *SD*; males: 4.22 ± 0.94; females: 4.30 ± 0.81; *p* = .706).

**Fig 3:**
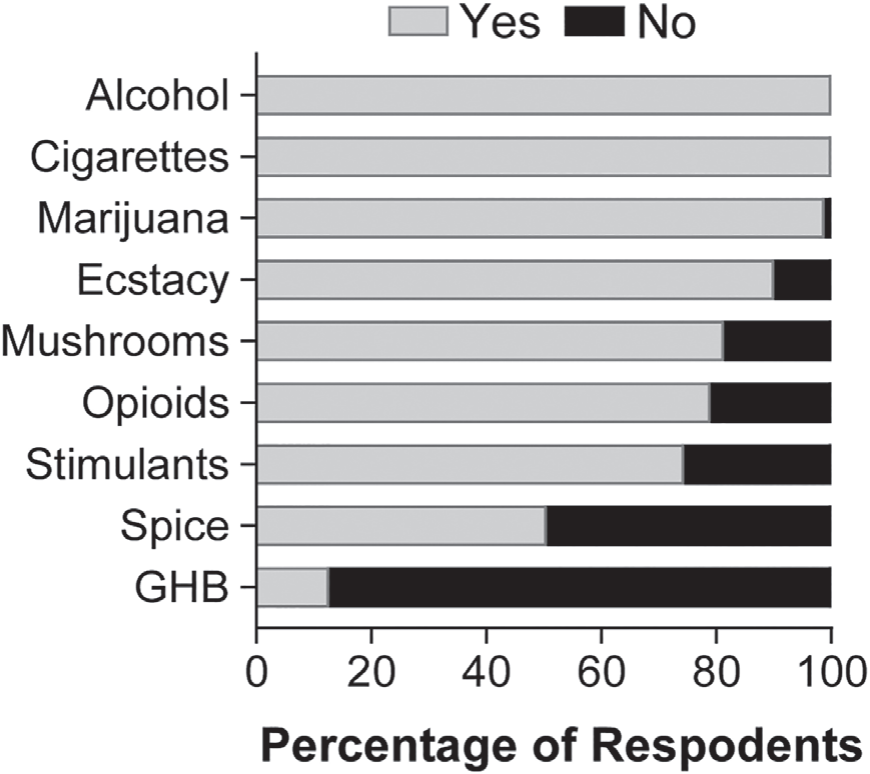
Reported familiarity with each drug before Drug Outreach, Promoting Awareness (DOPA) participation. Frequency distribution of familiarity with each drug. “Yes” represents high school students that had heard of the drug and “no” represents those that had not. For exact percentage values of unfamiliarity refer to Tables 1 or 2. *n* = 172 for all drugs except GHB (*n* = 110).

Our survey revealed that a majority of participating high school students had heard of the drugs alcohol, cigarettes, marijuana, ecstasy, mushrooms, and opioids, but fewer students had heard of the remaining drugs surveyed, including stimulants, spice/ K2, and Gamma‐hydroxybuterate (GHB) (Figure 3). Interestingly, the effect sizes of changes in harm risk and addictiveness ratings of these less commonly heard of drugs were greater than those of other drugs surveyed (*r* range 0.37–0.47) (Tables 1, 2), suggesting that prior familiarity with a substance may have affected our ability to change perceptions. To assess this, we next restricted analysis to subsets of high school students who had heard of a given drug (i.e., familiar) and those who had not heard of the drug (i.e., unfamiliar). When comparing pre‐ and postvisit ratings of all drugs combined for high school students who had reported being familiar with the drugs, Friedman tests revealed a significant increase in harmfulness, *χ* ^2^(1) = 4.50, *p* = .034, and addictiveness, *χ* ^2^(1) = 7.00, *p* = .008, ratings. These results largely mimicked the data reflective of all participants regardless of familiarity (Figure 2a). Wilcoxon paired *t*‐tests further revealed that harmfulness ratings for those familiar with a specific drug significantly increased for most drugs of abuse except for alcohol, cigarettes, and marijuana, although the effect sizes of significant comparisons were small (Table 3). Changes in addictiveness ratings followed a similar pattern, where ratings significantly increased for all drugs except for cigarettes (Table 4). Since only 14 out of 110 high school students (13%) had heard of GHB before DOPA, statistical tests for GHB were underpowered, and we therefore chose not to report the results for these students.

**Table 3:** Harmfulness Ratings for Familiar High School Students

		Mean rating (*SD*)			
Drug	*n*	Previsit	Postvisit	*p*	Effect size^a^
Alcohol	172	4.08 (0.94)	4.07 (0.94)	.987	0.00
Cigarettes	172	4.54 (0.79)	4.58 (0.75)	.596	0.03
Marijuana	170	3.18 (1.24)	3.22 (1.17)	.884	0.01
Ecstasy	155	4.41 (0.87)	4.61 (0.66)	.005	0.16
Mushrooms	140	4.11 (1.02)	4.38 (0.86)	.001	0.19
Opioids	136	3.62 (1.61)	4.15 (1.00)	<.001	0.26
Stimulants	128	3.16 (1.20)	3.93 (0.93)	<.001	0.35
Spice	87	3.71 (1.22)	4.59 (0.72)	<.001	0.43

a Effect size *r*.

**Table 4:** Addictiveness Ratings for Familiar High School Students

		Mean rating (*SD*)			
Drug	*n*	Previsit	Postvisit	*p*	Effect size^a^
Alcohol	172	4.01 (1.06)	4.27 (0.86)	<.001	0.20
Cigarettes	172	4.56 (0.78)	4.66 (0.67)	.162	0.08
Marijuana	170	3.60 (1.16)	3.82 (1.10)	.0420	0.11
Ecstasy	155	4.24 (0.97)	4.43 (0.82)	.0130	0.14
Mushrooms	140	3.86 (1.05)	4.23 (0.89)	<.001	0.22
Opioids	136	3.94 (1.22)	4.38 (0.90)	<.001	0.26
Stimulants	128	3.23 (1.19)	4.06 (0.94)	<.001	0.37
Spice	87	3.68 (1.21)	4.24 (0.96)	<.001	0.29

a Effect size *r*.

Similarly, for high school students who reported being unfamiliar with a given drug, ratings of all drugs combined for both harmfulness, *χ* ^2^(1) = 6.00, *p* = .014, and addictiveness, *χ* ^2^(1) = 6.00, *p* = .014, also significantly increased after DOPA participation. Wilcoxon paired *t*‐tests further showed increases in harmfulness and addictiveness perceptions for all drugs individually (Tables 5 and 6). Notably, the significant pre–post gains in both harm risk and addictiveness ratings for unfamiliar participants appeared much larger than those of the familiar participants (familiar *r* range 0.11–0.43; unfamiliar *r* range 0.37–0.56). Changes in ratings for high school students who were unfamiliar with marijuana are not reported given that only two students (1%) stated not knowing about marijuana prior to the visit, making statistical analysis untenable.

**Table 5:** Harmfulness Ratings for Unfamiliar High School Students

		Mean rating (*SD*)			
Drug	*n*	Previsit	Post‐isit	*p*	Effect size^a^
Ecstasy	17	2.88 (1.27)	4.59 (0.71)	<.001	0.56
Mushrooms	32	3.38 (1.16)	4.25 (1.02)	<.001	0.49
Opioids	36	3.25 (1.36)	4.19 (0.82)	<.001	0.38
Stimulants	44	2.95 (1.36)	4.05 (0.94)	<.001	0.43
Spice	85	3.24 (1.21)	4.53 (0.67)	<.001	0.50
GHB	96	3.23 (1.34)	4.38 (0.84)	<.001	0.44

a Effect size *r*.

**Table 6:** Addictiveness Ratings for Unfamiliar High School Students

		Mean rating (*SD*)			
Drug	*n*	Previsit	Postvisit	*p*	Effect size^a^
Ecstasy	17	2.76 (1.35)	4.24 (1.15)	<.001	0.53
Mushrooms	32	3.25 (1.22)	4.16 (0.95)	<.001	0.44
Opioids	36	3.00 (1.27)	4.36 (0.87)	<.001	0.51
Stimulants	44	3.16 (1.40)	4.16 (1.06)	<.001	0.41
Spice	85	3.09 (1.19)	4.32 (0.86)	<.001	0.46
GHB	96	3.26 (1.32)	4.16 (0.97)	<.001	0.37

a Effect size *r*.

### Survey Results: Open‐Ended Questions

To assess whether DOPA impacted attitudes about addiction treatment and what elements of the program resonated with high school students, we asked them several open‐ended questions. Answers to the addiction treatment question were classified into four categories subjectively determined based on the types of responses obtained. When asked what they would do if someone important to them asked about a treatment for addiction, there was a shift in responses after participating in DOPA, with a larger proportion of high school students stating they would refer the person to a medical professional, or addiction treatment center (Table 7).

**Table 7:** *Survey Question: What Would You Do If Someone Important to You Asked You About Treatment for An Addiction?*

			Participants (%)	
**Response category**			**Previsit**	**Postvisit**
Refer them to a medical professional, rehab, or addiction treatment center			72 (48%)	89 (60%)
Refer them to an adult (teacher, school counselor, or parent)			10 (7%)	3 (2%)
Help them myself by offering support			56 (38%)	54 (37%)
Nothing or “I don't know”			10 (7%)	2 (1%)

*Note. n* = 148.

**Fig 4:**
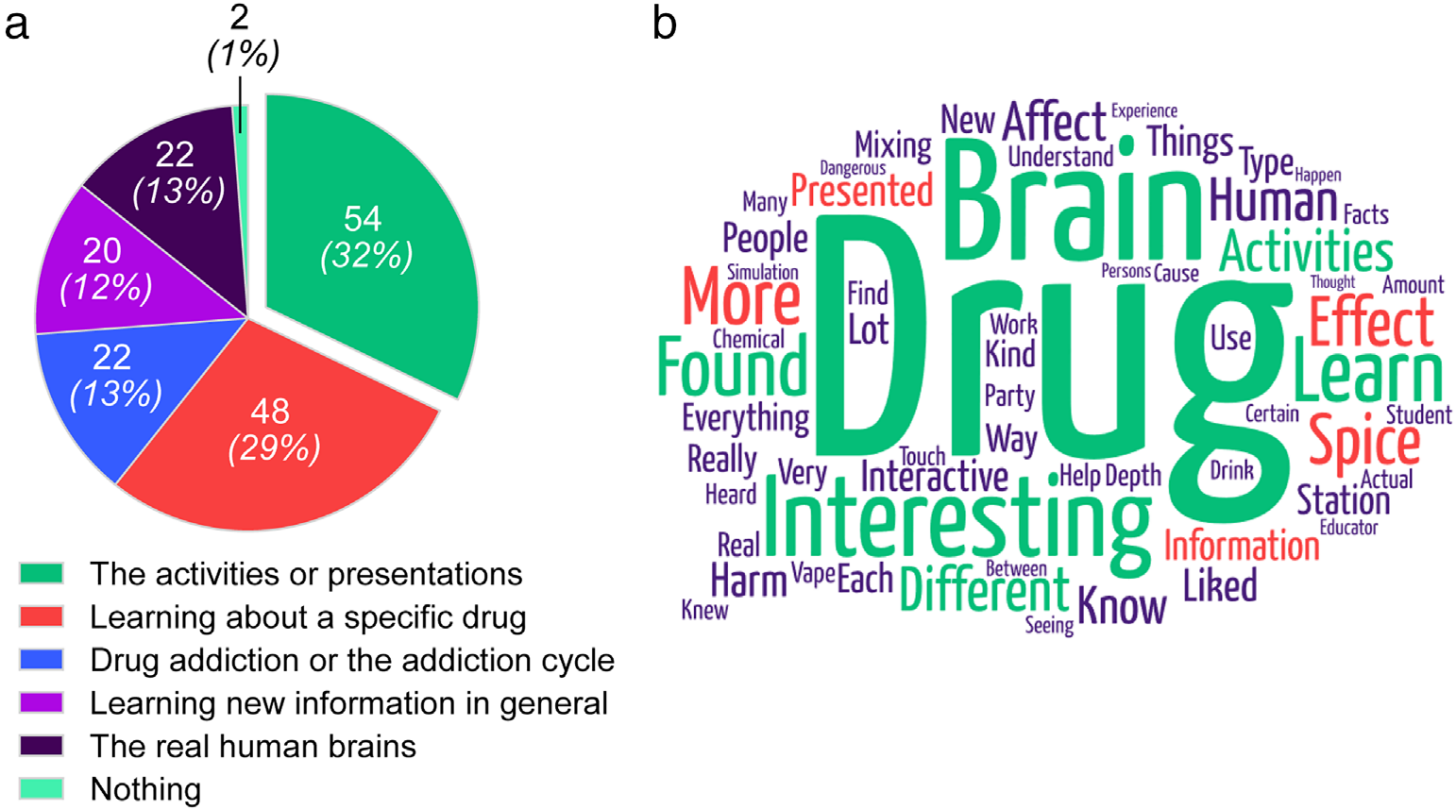
High school student comments and opinions about the Drug Outreach, Promoting Awareness (DOPA) visit. (a) Proportion of responses to open‐ended questions about what high school students found most interesting about the visit (*n* = 168). (b) Word cloud representing frequency of word use. The most commonly used words, defined as those used more than 15 times appear larger and are color coded in green. Moderately used words, defined as those used between 10 and 15 times, appear smaller and are red. The least frequent words, defined as those used fewer than 10 times, are the smallest and are purple. A total of 335 words were recorded from 168 survey responses. Total word frequency was 755.

Lastly, we asked high school students what they found particularly interesting about the visit. After partitioning the responses into six broad categories based on their content, we found that a vast majority of participants found the experience productive. More than half of them (61%) stated that the activities or presentations and learning about a particular drug or something specific about a drug was the most interesting element (Figure 4a).

A less biased view of responses to this open‐ended question can be represented qualitatively as a word cloud (https://wordart.com). In this format, word frequencies are rendered into font size, making more commonly used words larger. Figure 4b shows that the words “drug” and “brain” were used most frequently, which we attribute to the neuroscience focus of DOPA. Additional words such as “interesting, “learn,” and “activities” stand out suggesting that high school students found the experience productive. Overall, high school students highly appreciated the DOPA experience; when asked about how valuable they thought the visit was, most participants (93%) gave the program a 4 or 5 (1 = low value, 5 = high value; mean rating ± *SD*: 4.63 ± 0.68).

## DISCUSSION

Here we describe and evaluate DOPA, an undergraduate course and high school outreach program aimed at increasing awareness about the neurobiology, harm risks, and addictive potential of commonly used legal and illegal drugs. Preliminary survey results demonstrated that DOPA raised high school students' perceptions of the harm risks and the addictive potential of several drugs, particularly for those unfamiliar with certain substances. Overall, high school students appreciated the interactive activities and presentations. By formal training in teaching methodology, DOPA also allowed the undergraduate student instructors to build valuable skills in communicating science while connecting them to their local community.

After the DOPA school visits, our survey results showed that harmfulness ratings significantly increased for all drugs assessed except for alcohol, cigarettes, and marijuana. We posit that familiarity and prior beliefs or experience with these drugs prevented a shift in harmfulness perceptions. Indeed, alcohol and marijuana are the most widely used drugs amongst teenagers, with approximately 52% and 44% of 12th graders reporting use in the past year, respectively (Miech et al., 2019). Cigarette use is less prevalent but is higher than common illicit drugs; about 24% of 12th graders report having tried cigarettes in their lifetime.

Both alcohol and cigarettes were rated as more harmful than most other drugs prior to DOPA participation and these perceptions remained unchanged after the visit (Table 1). This is unsurprising given that both drugs are extensively covered by high school‐based health curricula and by substance abuse prevention programs, such as national antismoking campaigns (Farrelly et al., 2002; Miech et al., 2019). The lack of perceptual change can most plausibly be explained by a ceiling effect, meaning high school students already knew relevant risks or held longstanding beliefs about these substances. Our inability to shift harm risk attitudes about marijuana is expected given that in recent years adolescents view marijuana use as not harmful (Miech et al., 2019; Sarvet et al., 2018), particularly in states with legalization and medical marijuana, including California (D'Amico, Rodriguez, Tucker, Pedersen, & Shih, 2018; D'Amico, Tucker, Pedersen, & Shih, 2017; Ghosh et al., 2015).

Interestingly, although the harm risk of alcohol and marijuana did not change, addictiveness ratings for these drugs significantly increased, albeit with a small effect size (Table 2). Nevertheless, given that these addiction ratings did rise, it is possible that high school students may not necessarily grasp that most drugs are addictive. In the case of alcohol, the rise in addictiveness perceptions were driven by males, as they rated alcohol significantly more addictive after the visit, while the average addictiveness ratings for female high school students remained statistically unchanged. With respect to marijuana, participating students may have equated positive views with less risk of addiction and neglected the fact that it is not completely harmless. In line with our previsit findings, self‐report studies show that adolescents associate marijuana use with fewer long‐term health consequences and view the drug as less addictive compared to cigarettes or tobacco (Roditis & Halpern‐Felsher, 2015). Conversely, despite our efforts to convey that hallucinogens may pose certain harm risks to users, but mostly have low addictive risk (Canal & Murnane, 2017), addictiveness ratings of mushrooms significantly increased (Table 2). Given the increases in addictiveness perceptions for most drugs, it is possible that the high school students generalized lessons about addiction across all drugs, even to substances we emphasized as not having a high addictive potential.

Our ability to change overall attitudes about drugs was not disrupted by familiarity with various substances, but we did notice a difference in the magnitude of the effect. In particular, we noticed much larger gains in both harm risk and addictiveness perceptions of all drugs within the unfamiliar group compared to familiar high school students (Tables 3, 4, 5, 6), suggesting that DOPA might be more impactful for naïve adolescents. Indeed, the baseline perceptions were lower in unfamiliar compared to familiar participants, suggesting that a lack of knowledge of a particular drug may lead to a decreased perception of overall risk. This is especially salient as prior quality knowledge about drugs by adolescents has been shown to reduce drug use (Cuijpers, Jonkers, De Weerdt, & De Jong, 2002; Ramirez et al., 2004). Therefore, our program could serve as a simple, yet effective way to educate uninformed high school students.

After participating in DOPA, a larger proportion of high school students stated that they would refer a friend to a medical professional, or addiction treatment center (Table 7). Thus, the program may have increased adolescents' recognition of addiction as a medical condition or disorder requiring specialized treatment. Anecdotally, given their subject‐matter expertise and pedagogical training, undergraduate instructors may have been perceived by their audience as particularly knowledgeable and credible (Engelmann, Moore, Monica Capra, & Berns, 2012; Yeager, Dahl, & Dweck, 2018), which contributed to a shift in awareness towards legitimate addiction treatment.

Based on open‐ended responses, participating high school students found the hands‐on activities and presentations on certain drugs highly engaging (Figure 4a), suggesting that our interactive, drug‐specific approach was well‐received and memorable. Supporting this notion, the words “interesting,” “learn,” “activities,” and “presented” naturally appeared often in the responses (Figure 4b). Drug‐specific words like “spice” were also somewhat frequently used, indicating that perhaps this topic was particularly impactful.

### Limitations and Future Directions

Overall, although our preliminary findings suggest that the DOPA program was received positively and may change adolescent attitudes toward drugs, there are several limitations to our evaluation. First, we acknowledge that absent a formal longitudinal study, we cannot determine whether we are curtailing drug‐taking. Follow‐up evaluations of attitudes and drug use months or years after program participation would clarify whether the immediate, short‐term program impacts we measured in the survey translate to future drug use and attitudes. Second, we only evaluated the perceptions of students who willingly participated in the program and completed the pre‐ and postsurveys, which will necessarily introduce selection biases into our analyses. Indeed, voluntary survey respondents tend to have better health indicators (Cheung, Ten Klooster, Smit, de Vries, & Pieterse, 2017), which in our case might translate into skewed measures of program impact, as they are likely the ones already more aware of drug risks. Similarly, the strong female over‐representation might also distort the overall program impact as females are generally less likely to misuse drugs, but when they do, they exhibit enhanced drug use escalation and susceptibility to addiction (Becker & Hu, 2008). Regular reminders to students or mandatory surveys may reduce this sampling bias and in general increase overall response rates.

The program also lacked a control group, such as a group of high school students in a noninteractive, older adult‐instructed outreach program to clarify whether or not our interactive, peer‐led teaching approach played a causal role in shifting drug attitudes. Lastly, the effect sizes for pre–post drug perception comparisons were small to moderate for many drugs, potentially reflecting ceiling effects and/or potentially weak impacts on high school student attitudes due to numerous factors, including selection bias, or the fact that visits occurred only once, were brief (∼50 min), and covered a multitude of drugs.

Taken together, the DOPA program significantly changed high school student attitudes about drug risks and addiction for multiple drugs of abuse and is simple enough to be readily implemented in a broad range of high school classrooms. Moving forward, we plan to assess whether program outcomes are equivalent across schools, grades, ages, and socioeconomic backgrounds, as there could be meaningful differences which could further direct our outreach and educational efforts. We also continually update DOPA content; for instance, we now include vaping nicotine and cannabinoid products as a focus area. Given preliminary indications of success, as well as glowingly positive feedback from individual schools, we will soon initiate communications with LAUSD to formalize a system‐wide drug outreach collaboration.

In addition, DOPA functions as a rigorous university course that provides undergraduate students with the opportunity to master skills in science communication, writing, and teaching. Building these skills is essential for future career success in scientific research and medicine, and may inspire alternative career exploration (Brownell, Price, & Steinman, 2013; Edlow et al., 2007). Although we obtained basic course evaluation data, which confirmed that the undergraduates learned something valuable (1 = low, 9 = high; *n* = 23; mean rating ± *SD*: 8.94 ± 0.11) we did not tease apart the learning gains. As such, we soon plan to individually measure metrics such as communication skills and factual knowledge. Likewise, although we know that some students are reflecting on their career prospects (as evidenced from open ended comments such as *“After taking this class, I have considered changing my career path from a strictly pre‐health perspective to social work and engagement!”*), we need to assess long‐term outcomes, in particular how the program affected both their career choice and perceived success. By training undergraduate students to effectively convey scientific information to a high school audience, we foster an interactive learning environment that stretches beyond the ivory tower to impact the local community in an area of growing importance to society.

## Supporting information


**Supporting File S1** Supporting information

**Supporting File S2** Supporting information

**Supporting File S3** Supporting information

**Supporting File S4** Supporting information

**Supporting File S5** Supporting information
