# Supplementary material for: An Undergraduate Student‐Led Neuroscience Outreach Program Shows Promise in Shifting Teen Attitudes About Drugs
Source: Mind Brain Educ. 2020 Oct 4;14(4):387–99. doi: 10.1111/mbe.12261 (PMC7756680; doi:10.1111/mbe.12261)
Supplement: Supplementary file 1 — Supporting File S1 Supporting information [file MBE-14-387-s001.pdf]

UCLA DOPA Team

Drug Outreach, Promoting Awareness

BRAIN RESEARCH INSTITUTE UCLA

Drug Abuse and Society: Conveying Concepts to High School Students

Neuroscience 192C (4 units)

Course Instructors:

Chris Evans, PhD	MRL, room 2760<br>Tel: (310) 206-7883<br>e-mail: cevans@ucla.edu
Rafael Romero, PhD	Hershey Hall, room 220C<br>Tel: (310) 825-8922<br>e-mail: raromer@ucla.edu

TA:

Nina Lichtenberg	e-mail: ntlichtenberg@gmail.com

Course Description:

This course will prepare students to give an accurate, knowledgeable and age-appropriate lecture in the area of drug abuse to students at local high schools. The course is designed to: a) increase course participants' knowledge about drug abuse issues (including policy, biology and statistics) in our society, b) give students experience in preparing a presentation that would be suitable in a classroom setting, and c) provide UCLA students the opportunity and training to effectively translate their learned information of abused drugs to an at-risk population. The course is designed as a follow-up from Neuroscience C117/C277 (Drugs of Abuse: From Neurobiology to Policy and Education) which will provide students with basic knowledge about the neurobiology and pharmacology of different drugs of abuse and an overview of current addiction treatment approaches used and drug policy.

Prerequisites:

Course prerequisites include holding the status of Undergraduate Senior and having previously taken Neuroscience C177/C277. Graduate students at UCLA in an area that would enable them to be cognizant of drug abuse neurobiology and/or policy can also enroll.

Class Time and Location:

Mondays, 12:00 – 2:50 PM
Gonda 2303

Course Format, Assignments and Grading:

Class meeting will feel more like workshops than lectures. The idea is to create an open environment where students and instructors can freely discuss concepts, clarify misconceptions and exchange ideas. Different resources will be made available prior to and during the class meetings to help foster these discussions. As such, students are expected to review the material before coming to class and to engage in scholarly discourse in every class as they work on their drug presentations.

Grading will be based on a series of activities all leading to the generation of an informative and engaging school presentation about different drug categories. Students will work in pairs, and will choose one of four broad drug categories (see below). Presentations will be crafted to introduce those categories, providing both the biological/clinical importance as well as some social and historical context. Within each drug category presentation, each group will choose and discuss two representative drugs to provide precise examples of their mode of action and societal impact. Each student will prepare an informative trifold pamphlet of one of the two drugs to be presented at the schools. An integral component of these presentations will be the generation of hands-on activities that the high school students can use to review and practice the concepts were taught to them. Please see instructions below for details on how to put together the different assignments.

At the end of the course the students will be asked to give us a copy of all their work to be compiled and ultimately used by local schools as a pedagogical tool. Due credit will always be given to the students unless he/she prefers to remain anonymous.

This course will be letter graded on a straight scale: 98-100% → A+, 93-97% → A, 90-92% → A-, 87-89% → B+, 83-86% → B, 80-82% → B-, 77-79% → C+, 73-76% → C, 70-72 → C-, 55-69 → D, 0-54% → F; with a 70% (C) considered a passing grade. Please keep in mind that there are no curves

in this course so each student earns their grade based on the scale above regardless of what the class grade distribution looks like. The grading structure is as follows:

Weekly participation →	10%		
Final draft of pamphlet →	10%	Lesson plans, drug →	10%
First PowerPoint draft &		Drug station model/activity →	10%
Rehearsal of presentation →	10%	In-school presentation →	20%
Final PowerPoint draft &		In-school station management →	20%
Dress rehearsal of presentation →	10%		

Class Schedule:

Week 1<br>(Apr. 2)	Introduction to course.<br>General discussion on presentations to K-12.<br>Group selection.<br>Assignment of drug categories for presentations, hands-on stations and pamphlets.<br>Review last year's survey questions and discussion.
Week 2<br>(Apr. 9)	Bring pdf of rough draft of pamphlet to class for critique.<br>Bring ideas for drug stations, group discussion.<br>Upload rough draft of PowerPoint presentation (Friday, Apr. 13)
Week 3<br>(Apr. 16)	Turn in detailed lesson plans for drug stations.<br>Provide budget for station materials if necessary.<br>Drug presentations: First rehearsals.
Week 4<br>(Apr. 23)	Drug presentations: First rehearsals (continued).<br>Practice run of drug station activity. Bring models if available.
Week 5<br>(Apr. 30)	Final draft of pamphlets due.<br>Drug presentations: Dress rehearsals (full presentation + activity)

Week 6<br>(May 7)	Check and fold printed pamphlets<br>Drug presentations: Dress rehearsals (full presentation + activity) [continued]<br>Upload final draft of PowerPoint presentation (Friday, May 11)
Week 7<br>(May 14)	High School visit #1
Week 8<br>(May 21)	High School visit #2
Week 9<br>(May 28)	**Memorial Day Holiday**
Week 10<br>(Jun. 4)	High School visit #3
Week 11<br>(Jun. 11-15)	Pizza and debriefing the school visits (Time & Day TBD)

Instructions for Drug Presentations

Students will be assigned to groups and assigned one of the following drug categories (see below). Each group will prepare a short, yet informative presentation on their drug category as well as a creative and engaging "station" to teach about their assigned drug category. Each group presentation should be no longer than 15 minutes while the station activity should take between 5-10 minutes to complete. Both members of the group are expected to present and the work should be shared equally. During each school visit, one group will give a presentation on their assigned drug category, while the other groups will set up their drug specific stations.

PowerPoint presentations will be limited to have no more than 15 slides. Importantly, each slide should have minimal text and only contain *one* idea or concept. The presentation will consist of three parts: 1) a general introduction, 2) introduction to drug category, and 3) example drugs. The general introductory section can include topics like the anatomy of the brain, function of neurons and synapses, reward pathways and addiction. The drug category section should introduce the actual drugs, their mechanism(s) of action and social implications. Think of this section as the "why should we care about these drugs" section. The last section should focus on two representative drugs for your category. Include information on how and why the drugs are abused and what are the biological or social consequences.

You will realize that given the strict space and time constraints you will not be able to provide a comprehensive account of your drug category. Remember that your job is to raise drug awareness, not to turn your audience into drug addiction experts! This will force you to simply highlight the critical concepts during your presentation and to move other important details into your hands-on activity. Indeed an effective presentation will invite the students to visit your station to learn more about your drug category.

Drug categories:

1. Prescription drugs (opioids and psychostimulants)
2. Legal drugs (alcohol and nicotine)
3. Cannabinoids (marijuana and spice)
4. Party drugs (date-rape drugs, rave drugs)

Instructions for Written Assignments

Drug information pamphlets

As part of the school visits, we will provide the students with informational pamphlets that they can use as a reference and which tells them how and where to find more information on the particular drugs you focus on individually. The pamphlet should be brief (no more than one page, front and back, for each drug) and should summarize your drug presentations. As such, it should include general information on your drug class as well as specific information on your example drugs. Be sure to also include links to vetted on-line resources that students can use to find additional information. Feel free to include images, pictures, graphs and any other visual aids that will make your pamphlet more user friendly. Pamphlets should be formatted into three columns so that the page can be folded appropriately. Examples of previous pamphlets will be shown in class. They will be graded for content and age-appropriateness, and students will be asked to address any comments before printing the pamphlets.

Station lesson plans

The station lesson plans should include all the relevant information that you will need to both create the station and to prepare yourself to answer questions from the high school students. The station should convey all the information that is in the presentation, and should be creative.
