# Supplementary material for: An Undergraduate Student‐Led Neuroscience Outreach Program Shows Promise in Shifting Teen Attitudes About Drugs
Source: Mind Brain Educ. 2020 Oct 4;14(4):387–99. doi: 10.1111/mbe.12261 (PMC7756680; doi:10.1111/mbe.12261)

Do amphetamines (like Adderall) make you **smarter**?

NOPE!

In 2013,

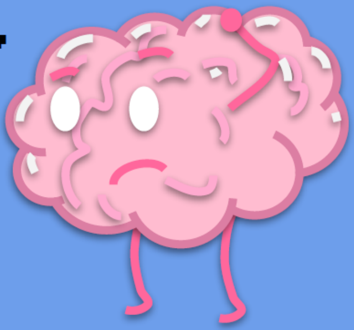

7.4%

of high schoolers took Adderall.

Many believed it would improve their grades...

Both Adderall and a harmless sugar pill (placebo) were shown to improve grades equally!

It's all in your head!

LEARN MORE HERE!

DrugAbuse.gov

YouTube

"Your Brain On Adderall"

"How Does Adderall Work?"

"STUDY DRUGS"

Prescription Amphetamines

(Adderall)

By: Blaine "brain" Eldred

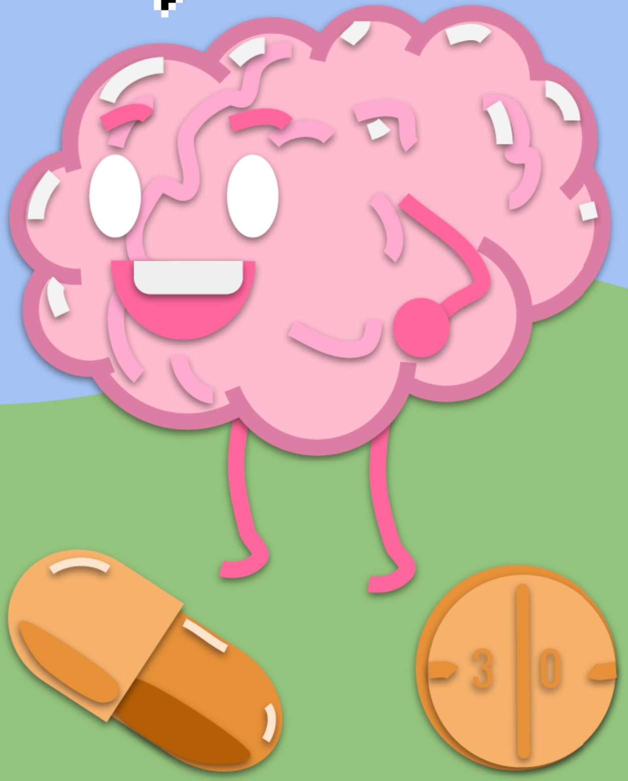

UCLA DOPA Team
Drug Outreach, Promoting Awareness

WHAT ARE AMPHETAMINES?

Amphetamines are a type of **prescription medication** used to treat:

- ADHD
- Narcolepsy
- Depression

Amphetamines, are **STIMULANTS** so they increase **focus** and **energy**.

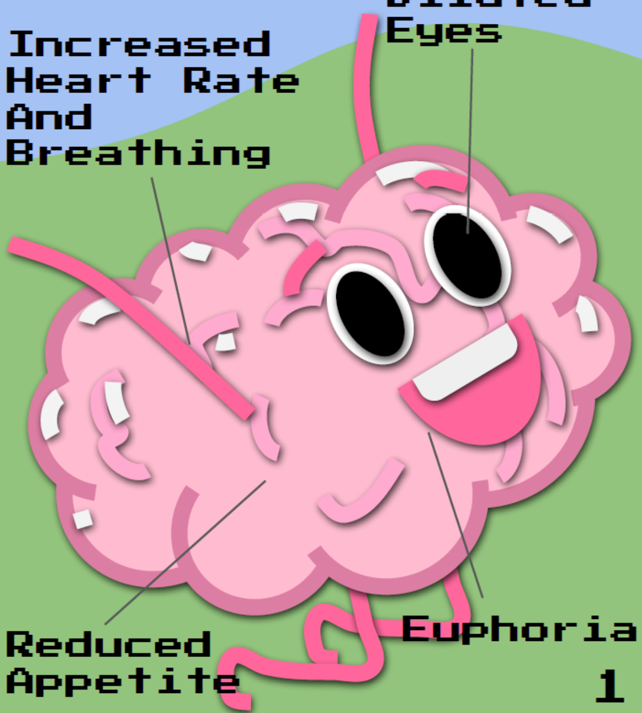

IS ABUSING AMPHETAMINES DANGEROUS?

YES! Amphetamines can be **addictive**. The more you take, the more you *NEED*.

It's also possible to **overdose** on amphetamines. An overdose is a **life-threatening medical emergency**!

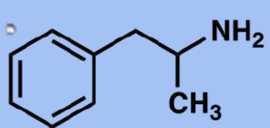

Adderall

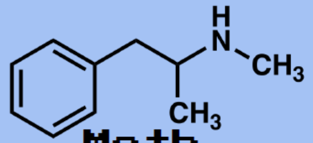

Meth

Can you spot the difference?

There is a ***CRASH*** after the **high**. It feels like the exact opposite of the high:

Ouch

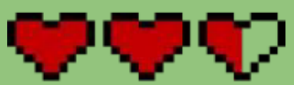

Such Sad

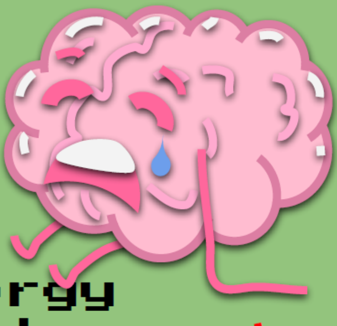

- Low Energy
- Difficulty Focusing
- Depression

Much Crash

Studying Tips!

Instead of taking amphetamines **illegally** (w/o prescription), Try these:

- Turn off your cell phone
- Lower volume of music
- Close social media websites

These weapons of mass distraction are sure to make focusing difficult.

Cramming for an exam? Try Coffee, Tea, or Chocolate for a safer boost of caffeine energy!

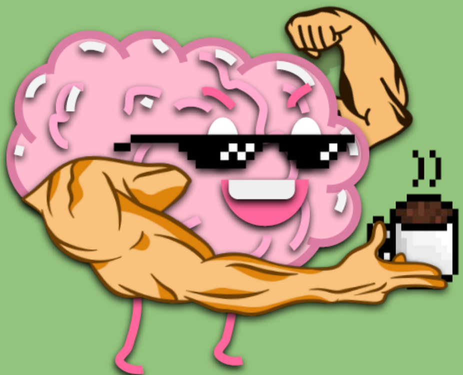

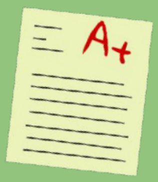

Supplement: Supplementary file 2 — Supporting File S2 Supporting information [file MBE-14-387-s002.pdf]
