# Supplementary material for: An Undergraduate Student‐Led Neuroscience Outreach Program Shows Promise in Shifting Teen Attitudes About Drugs
Source: Mind Brain Educ. 2020 Oct 4;14(4):387–99. doi: 10.1111/mbe.12261 (PMC7756680; doi:10.1111/mbe.12261)
Supplement: Supplementary file 3 — Supporting File S3 Supporting information [file MBE-14-387-s003.docx]

† Title 1 school (school receiving federal funds for Title 1 students; at least 40% of students come from families that qualify as low-income under the United States Census definitions)

‡ Schools in which over 50% of total students are eligible to participate in the Free Lunch and Reduced-Price Lunch Programs under the National School Lunch Act of 1946.
§ Data are from the 2018-2019 school year (except for the International School of Los Angeles - Burbank, for which 2017-2018 data are used)

|  |  |  | **Enrollment by race/ ethnicity**^§^ | | | | | | |
| --- | --- | --- | --- | --- | --- | --- | --- | --- | --- |
| **School name** | **Type of school** | **Total number of students in school**^§^ | American Indian | Asian | Black/ African American | Hispanic/ Latino | Pacific Islander | White | Two or more races |
| Alexander Hamilton Senior High^†‡^ | Public | 2,602 | 0.4% | 5.2% | 26.1% | 51.8% | 0.2% | 16.2% | 0.0% |
| Fairfax Senior High^†‡^ | Public | 1,827 | 0.0% | 18.8% | 15.3% | 58.5% | 0.3% | 6.8% | 0.2% |
| International School of Los Angeles - Burbank | Private | 330 | 0.0% | 3.9% | 1.8% | 10.3% | 0.0% | 74.8% | 9.1% |
| New West Charter | Public | 887 | 0.6% | 8.0% | 5.7% | 25.8% | 0.1% | 52.1% | 7.6% |
| Theodore Roosevelt Senior High^†‡^ | Public | 1,278 | n/a | 0.5% | 0.3% | 98.7% | n/a | 0.5% | n/a |
| University High School Charter^†‡^ | Public | 1,511 | 0.0% | 8.7% | 25.0% | 53.0% | 0.4% | 12.8% | 0.0% |

**Demographic summary of high schools within the Greater Los Angeles Area visited by Drug Outreach, Promoting Awareness (DOPA) during the 2016-2018 school years.** DOPA visited high school students in grades 9-12 (~14-18-year-olds). The race/ ethnic profile, total number of students, Title 1 status, and free lunch eligibility data were obtained from the National Center for Education Statistics Common Core of Data (CCD) and the Private School Universe Survey (PSS) via publicly avaliable school databases provided by the National Center for Education Statistics (Public schools: <https://nces.ed.gov/ccd/schoolsearch/>; Private schools: <https://nces.ed.gov/surveys/pss/privateschoolsearch/>). A majority of the survey data in the main text (91.3%; 157/172 responses) were collected from high school students in public schools.
