# Supplementary material for: An Undergraduate Student‐Led Neuroscience Outreach Program Shows Promise in Shifting Teen Attitudes About Drugs
Source: Mind Brain Educ. 2020 Oct 4;14(4):387–99. doi: 10.1111/mbe.12261 (PMC7756680; doi:10.1111/mbe.12261)
Supplement: Supplementary file 4 — Supporting File S4 Supporting information [file MBE-14-387-s004.pdf]

Pre Survey Spring 2017

* Required

These questions will help us group your answers and match your responses on the pre-visit survey to those on the post-visit survey. Please remember your answers to this section and use the same answers when you fill out the post-visit survey.

1. **What is the name of your school?** *

2. **Gender** *

   Mark only one oval.

   - Female
   - Male
   - Other: ______

3. **What are the first three letters of your mother's name?** *

4. **What is your favorite color?** *

5. **What is your favorite number?** *

Please remember answers to these questions for future use!

Untitled Section

Marijuana

Other names: Weed, Pot, Hash

6. **Have you heard of this drug?** *

   Mark only one oval.

   - Yes
   - No

7. **How common is this drug among your peers?** *
Mark only one oval.

	1	2	3	4	5	
Not common	◯	◯	◯	◯	◯	Very common

8. **How harmful do you think this drug is?** *
Mark only one oval.

	1	2	3	4	5	
Not harmful	◯	◯	◯	◯	◯	Very harmful

9. **How addictive is this drug?** *
Mark only one oval.

	1	2	3	4	5	
Not addictive	◯	◯	◯	◯	◯	Very addictive

Spice/K2

Other names: synthetic marijuana, fake/legal pot,

10. **Have you heard of this drug?** *
Mark only one oval.

◯ Yes
◯ No

11. **How common is this drug amongst your peers?** *
Mark only one oval.

	1	2	3	4	5	
Not common	◯	◯	◯	◯	◯	Very common

12. **How harmful do you think this drug is?** *
Mark only one oval.

	1	2	3	4	5	
Not harmful	◯	◯	◯	◯	◯	Very harmful

13. **How addictive is this drug?** *
Mark only one oval.

	1	2	3	4	5	
Not addictive	⬭	⬭	⬭	⬭	⬭	Very addictive

Alcohol

14. **Have you heard of this drug?** *
Mark only one oval.

- ⬭ Yes
- ⬭ No

15. **How common is this drug amongst your peers?** *
Mark only one oval.

	1	2	3	4	5	
Not common	⬭	⬭	⬭	⬭	⬭	Very common

16. **How harmful do you think this drug is?** *
Mark only one oval.

	1	2	3	4	5	
Not harmful	⬭	⬭	⬭	⬭	⬭	Very harmful

17. **How addictive is this drug?** *
Mark only one oval.

	1	2	3	4	5	
Not addictive	⬭	⬭	⬭	⬭	⬭	Very addictive

ADHD medication
Other names: Adderall, Dexedrine, Dextrostat, amphetamine

18. **Have you heard of this drug?** *
Mark only one oval.

- ⬭ Yes
- ⬭ No

19. **How common is this drug amongst your peers?** *
Mark only one oval.

	1	2	3	4	5	
Not common	◯	◯	◯	◯	◯	Very common

20. **How harmful do you think this drug is?** *
Mark only one oval.

	1	2	3	4	5	
Not harmful	◯	◯	◯	◯	◯	Very harmful

21. **How addictive is this drug?** *
Mark only one oval.

	1	2	3	4	5	
Not addictive	◯	◯	◯	◯	◯	Very addictive

Opiate Pain Killers

22. **Have you heard of this drug?** *
Mark only one oval.

◯ Yes
◯ No

23. **How common is this drug amongst your peers?** *
Mark only one oval.

	1	2	3	4	5	
Not common	◯	◯	◯	◯	◯	Very common

24. **How harmful do you think this drug is?** *
Mark only one oval.

	1	2	3	4	5	
Not harmful	◯	◯	◯	◯	◯	Very harmful

25. **How addictive is this drug?** *
Mark only one oval.

	1	2	3	4	5	
Not addictive	◯	◯	◯	◯	◯	Very addictive

Cigarettes/Nicotine

26. **Have you heard of this drug?** *
Mark only one oval.

- Yes
- No

27. **How common is this drug amongst your peers?** *
Mark only one oval.

	1	2	3	4	5	
Not common						Very common

28. **How harmful do you think this drug is?** *
Mark only one oval.

	1	2	3	4	5	
Not harmful						Very harmful

29. **How addictive is this drug?** *
Mark only one oval.

	1	2	3	4	5	
Not addictive						Very addictive

Mushrooms/ Psilocybin

30. **Have you heard of this drug?** *
Mark only one oval.

- Yes
- No

31. **How common is this drug amongst your peers?** *
Mark only one oval.

	1	2	3	4	5	
Not common						Very common

32. **How harmful do you think this drug is?** *
Mark only one oval.

	1	2	3	4	5	
Not harmful	◯	◯	◯	◯	◯	Very harmful

33. **How addictive is this drug?** *
Mark only one oval.

	1	2	3	4	5	
Not addictive	◯	◯	◯	◯	◯	Very addictive

Ecstacy/Molly

34. **Have you heard of this drug?** *
Mark only one oval.

◯ Yes
◯ No

35. **How common is this drug amongst your peers?** *
Mark only one oval.

	1	2	3	4	5	
Not common	◯	◯	◯	◯	◯	Very common

36. **How harmful do you think this drug is?** *
Mark only one oval.

	1	2	3	4	5	
Not harmful	◯	◯	◯	◯	◯	Very harmful

37. **How addictive is this drug?** *
Mark only one oval.

	1	2	3	4	5	
Not addictive	◯	◯	◯	◯	◯	Very addictive

Skip to question 38.

GHB

Other names: G, liquid G

38. **Have you heard of this drug?** *
Mark only one oval.

- Yes
- No

39. **How common is this drug amongst your peers?** *
Mark only one oval.

	1	2	3	4	5	
Not common						Very common

40. **How harmful do you think this drug is?** *
Mark only one oval.

	1	2	3	4	5	
Not harmful						Very harmful

41. **How addictive is this drug?** *
Mark only one oval.

	1	2	3	4	5	
Not addictive						Very addictive

Untitled Section

42. **Do you have any questions/drugs you would like to learn about?**

43. **What would you do if someone important to you asked you about treatment for an addiction?**

44. **How would you rate your interest in a visit from the UCLA Drug Outreach: Promoting Awareness team (DOPAteam)?**
Mark only one oval.

	1	2	3	4	5	
Low Interest	◯	◯	◯	◯	◯	High Interest

45. **How would you rate your interest in the science behind drug addiction?** *
Mark only one oval.

	1	2	3	4	5	
Low interest	◯	◯	◯	◯	◯	High interest

Powered by
Google Forms
