# Supplementary material for: An Undergraduate Student‐Led Neuroscience Outreach Program Shows Promise in Shifting Teen Attitudes About Drugs
Source: Mind Brain Educ. 2020 Oct 4;14(4):387–99. doi: 10.1111/mbe.12261 (PMC7756680; doi:10.1111/mbe.12261)
Supplement: Supplementary file 5 — Supporting File S5 Supporting information [file MBE-14-387-s005.pdf]

Post Visit Survey Spring 2017

* Required

These questions will help us group your answers and match your responses on the pre-visit survey to those on the post-visit survey. Please try to put the same answers for this section as you did during the pre-visit survey.

1. **What is the name of your school?** *

2. **Gender** *

   Mark only one oval.

   - Female
   - Male
   - Other:

3. **What are the first three letters of your mother's name?** *

4. **What is your favorite color?** *

5. **What is your favorite number?**

Untitled Section

Marijuana

Other names: Weed, pot, hash

6. **How harmful do you think this drug is?** *

   Mark only one oval.

	1	2	3	4	5	
Not harmful						Very harmful

7. **How addictive do you think this drug is?** *
   Mark only one oval.

	1	2	3	4	5	
Not addictive	◯	◯	◯	◯	◯	Very addictive

8. **How valuable was the information you learned about this drug from today's presentation?** *
   Mark only one oval.

	1	2	3	4	5	
Not valuable	◯	◯	◯	◯	◯	Very valuable

Spice/K2

Other names: Synthetic marijuana, "fake pot"

9. **How harmful do you think this drug is?** *
   Mark only one oval.

	1	2	3	4	5	
Not harmful	◯	◯	◯	◯	◯	Very harmful

10. **How addictive do you think this drug is?** *
    Mark only one oval.

	1	2	3	4	5	
Not addictive	◯	◯	◯	◯	◯	Very addictive

11. **How valuable was the information you learned about this drug from today's presentation?** *
    Mark only one oval.

	1	2	3	4	5	
Not valuable	◯	◯	◯	◯	◯	Very valuable

Alcohol

12. **How harmful do you think this drug is?** *
    Mark only one oval.

	1	2	3	4	5	
Not harmful	◯	◯	◯	◯	◯	Very harmful

13. **How addictive do you think this drug is?** *
Mark only one oval.

	1	2	3	4	5	
Not addictive	◯	◯	◯	◯	◯	Very addictive

14. **How valuable was the information you learned about this drug from today's presentation?** *
Mark only one oval.

	1	2	3	4	5	
Not valuable	◯	◯	◯	◯	◯	Very valuable

ADHD Medications

Other names: Adderall, Dexedrine, Dextrostat, amphetamine

15. **How harmful do you think this drug is?** *
Mark only one oval.

	1	2	3	4	5	
Not harmful	◯	◯	◯	◯	◯	Very harmful

16. **How addictive do you think this drug is?** *
Mark only one oval.

	1	2	3	4	5	
Not addictive	◯	◯	◯	◯	◯	Very addictive

17. **How valuable was the information you learned about this drug from today's presentation?** *
Mark only one oval.

	1	2	3	4	5	
Not valuable	◯	◯	◯	◯	◯	Very valuable

Opiate Pain Killers

18. **How harmful do you think this drug is?** *
Mark only one oval.

	1	2	3	4	5	
Not harmful	◯	◯	◯	◯	◯	Very harmful

19. **How addictive do you think this drug is?** *
Mark only one oval.

	1	2	3	4	5	
Not addictive	◯	◯	◯	◯	◯	Very addictive

20. **How valuable was the information you learned about this drug from today's presentation?** *
Mark only one oval.

	1	2	3	4	5	
Not valuable	◯	◯	◯	◯	◯	Very valuable

Cigarettes/Nicotine

21. **How harmful do you think this drug is?** *
Mark only one oval.

	1	2	3	4	5	
Not harmful	◯	◯	◯	◯	◯	Very harmful

22. **How addictive do you think this drug is?** *
Mark only one oval.

	1	2	3	4	5	
Not addictive	◯	◯	◯	◯	◯	Very addictive

23. **How valuable was the information you learned about this drug from today's presentation?** *
Mark only one oval.

	1	2	3	4	5	
Not valuable	◯	◯	◯	◯	◯	Very valuable

Mushrooms/Psilocybin

24. **How harmful do you think this drug is?** *
Mark only one oval.

	1	2	3	4	5	
Not harmful	◯	◯	◯	◯	◯	Very harmful

25. **How addictive do you think this drug is?** *
Mark only one oval.

	1	2	3	4	5	
Not addictive	◯	◯	◯	◯	◯	Very addictive

26. **How valuable was the information you learned about this drug from today's presentation?** *
Mark only one oval.

	1	2	3	4	5	
Not valuable	◯	◯	◯	◯	◯	Very valuable

Ecstasy/Molly

27. **How harmful do you think this drug is?** *
Mark only one oval.

	1	2	3	4	5	
Not harmful	◯	◯	◯	◯	◯	Very harmful

28. **How addictive do you think this drug is?** *
Mark only one oval.

	1	2	3	4	5	
Not addictive	◯	◯	◯	◯	◯	Very addictive

29. **How valuable was the information you learned about this drug from today's presentation?** *
Mark only one oval.

	1	2	3	4	5	
Not valuable	◯	◯	◯	◯	◯	Very valuable

GHB

Other names: G, liquid G

30. **How harmful do you think this drug is?** *
Mark only one oval.

	1	2	3	4	5	
Not harmful	◯	◯	◯	◯	◯	Very Harmful

31. **How addictive do you think this drug is? ***
Mark only one oval.

	1	2	3	4	5	
Not addictive	⬭	⬭	⬭	⬭	⬭	Very addictive

32. **How valuable was the information you learned about this drug from today's presentation? ***
Mark only one oval.

	1	2	3	4	5	
Not valuable	⬭	⬭	⬭	⬭	⬭	Very valuable

Untitled Section

33. **What did you find particularly interesting about today's visit? ***

____

34. **What would you do if someone important to you asked you about treatment for an addiction? ***

____
____
____
____
____

35. **How valuable was your visit from the UCLA Drug Outreach, Promoting Awareness team (DOPAteam)? ***
Mark only one oval.

	1	2	3	4	5	
Not valuable	⬭	⬭	⬭	⬭	⬭	Very valuable

36. **How would you rate your interest in the science behind drug addiction? ***
Mark only one oval.

	1	2	3	4	5	
Low interest	⬭	⬭	⬭	⬭	⬭	High interest

Powered by
Google Forms
